# Chinese Tobacco Industry Promotional Activity on the Microblog Weibo

**DOI:** 10.1371/journal.pone.0099336

**Published:** 2014-06-10

**Authors:** Fan Wang, Pinpin Zheng, Dongyun Yang, Becky Freeman, Hua Fu, Simon Chapman

**Affiliations:** 1 Key Laboratory of Public Health Safety, Ministry of Education, School of Public Health, Fudan University, Shanghai, China; 2 School of Statistics and Management, Shanghai University of Finance and Economics, Shanghai, China; 3 School of Public Health, University of Sydney, Sydney, Australia; Newcastle University, United Kingdom

## Abstract

**Background:**

Although China ratified the WHO Framework Convention on Tobacco Control [FCTC] in 2005, the partial ban on tobacco advertising does not cover the internet. Weibo is one of the most important social media channels in China, using a format similar to its global counterpart, Twitter. The Weibo homepage is a platform to present products, brands and corporate culture. There is great potential for the tobacco industry to exploit Weibo to promote products.

**Methods:**

Seven tobacco industry Weibo accounts that each had more than 5000 fans were selected to examine the content of Weibos established by tobacco companies or their advertising agents.

**Results:**

Of the 12073 posts found on the seven accounts, 92.3% (11143) could be classified into six main themes: traditional culture, popular culture, social and business affairs, advertisement, public relations and tobacco culture. Posts under the theme of popular culture accounted for about half of total posts (49%), followed by ‘advertisement’ and ‘tobacco culture’ (both at 12%), ‘traditional culture’ and ‘public relations’ (both at 11%), and finally ‘social and business affairs’ (5%). 33% of posts included the words ‘cigarette’ or ‘smoking’ and 53% of posts included the tobacco brand name, indicating that tobacco companies carefully construct the topic and content of posts.

**Conclusions:**

Weibo is an important new online marketing tool for the Chinese tobacco industry. Tobacco industry use of Weibo to promote brands and normalize smoking subverts China's ratification of the WHO FCTC. Policy to control tobacco promotion needs reforming to address this widespread circumvention of China's tobacco advertising ban.

## Introduction

Guidelines for the implementation of Article 13 of the WHO Framework Convention on Tobacco Control [FCTC] emphasize that a comprehensive ban on tobacco advertising should cover ‘any form of commercial communication, recommendation or action with the aim, effect or likely effect of promoting a tobacco product or tobacco use either directly or indirectly’ [1]. China ratified the WHO FCTC in 2005. However, the WHO FCTC has not been well implemented in China and tobacco brands continue to be promoted directly and indirectly through a variety of channels, especially via websites [2], [3].

The current tobacco provisions of the Advertisement Law of the People's Republic of China prohibit tobacco advertising in movies, radio, television, newspapers, journals, magazines, waiting rooms, cinemas, theatres, conference halls, stadiums, and gyms [4]. Previous research has shown that when governments ban or curtail tobacco advertising in traditional media, the tobacco industry pursues its promotional ambitions through new media [5], [6], [7], [8], [9].

Internet promotions present a massive and largely unregulated marketing channel for the tobacco industry with online social media being particularly important. Social media also offer the tobacco industry a powerful and efficient channel for rapidly countering the denormalising strategies and policies of tobacco control [10]. The tobacco industry has realized that social media are unparalleled new marketing platforms to influence customers, especially youth, and establish positive brand and company images. British American Tobacco employees were shown to be energetically promoting BAT and BAT brands on Facebook [11]. The Camel brand was promoted through interactive activities such as participatory packaging design via a corporate website [7], [9].

Weibo is one of China's most important social networking channels. ‘Weibo’ is the Chinese phonetic translation for ‘Micro Blog’, and is the Chinese counterpart to Twitter, which is blocked in China. As with Twitter, Weibo users open an account and can then post up to 140-characters. Users can also include web links and emoticons or attach images, music, or video files to each of their posts. Users can either make original posts or re-share content posted by other Weibo users. Weibo posting has become the most popular activity among Chinese internet users [12]. Followers of one's Weibo account are called ‘fans’. Fans receive Weibo posts and can choose to further disseminate them to their own Weibo network [13]. Posts on Weibo can also include a ‘topic tag’ so that users can search for content of interest to them. Weibo commenced operation in August 2009 and grew with phenomenal speed. By the end of June 2013, of 591 million internet users in China, 330 million had Weibo accounts [14], which lead to its most important role in the social media marketing platform [12].

In 2013, the Chinese website (http://www.tobaccochina.com), which is a professional website supported by Chinese tobacco industries with the most authoritative information on available tobacco products [15], [16], published an article titled ‘How to use Weibo in tobacco marketing' stating (translated from Chinese):

*‘The open platform of Weibo, is becoming an integral part of a new marketing channel for tobacco industries. As a new communication channel, Weibo significantly reduces the costs of promotion and advertising…. it can break through the current limitation of tobacco advertisement and play an important role in advertisement and promotion….The most popular information or activities of Weibo should be designed to reflect the needs of the consumers, as well as their attitudes and value orientation…. It is helpful to establish the positive image of tobacco industry among the public……Most efforts should be made to figure out most effective strategy in Weibo marketing…’* [17]

Up to the time of this study, the tobaccochina website mentioned Weibo marketing strategies more than other avenues due to its leading role in social media [18]–[20].

To date, there have been no published studies on internet-based tobacco marketing and promotion in China. This study describes and analyzes the thematic content of Weibo posts by Chinese tobacco companies in order to explore how they seek to influence their target audiences. Such studies can provide evidence to inform evaluation of the implementation of the WHO FCTC Article 13 and assist in establishing a more comprehensive policy banning all forms of tobacco advertising.

## Method

A thematic content analysis was conducted to assess how tobacco companies promote their products on Weibo [21], [22]. Error! Hyperlink reference not valid.We opened a Weibo account with Sina Weibo (www.weibo.com), the most popular micro blog operator in China, with 86.6% of China's Weibo market based on browsing time [23]. Sina Weibo has over 300 million registered users, and is still rapidly growing. It is estimated that there is an average of over 100 million posts per day made via Sina Weibo [24].

To determine which tobacco companies and brands to search for on Weibo, we obtained a list of current tobacco companies and cigarette brands in China from the tobacco marketing website ‘smoking happiness’ (www.yanyue.cn) [25]. Twenty nine tobacco companies were identified, together selling 98 cigarette brands with thousands of variants.

We then used the names of tobacco companies and cigarette brands as key words in Sina Weibo's search engine to find tobacco companies' Weibo accounts. Weibo accounts that included tobacco content but were operated by private citizens were not included in this study. In total, there were 33 Chinese tobacco company owned Weibo accounts. We ranked these 33 Weibo accounts by their number of fans. In order to make our sample size manageable, we decided to further examine only the top ten most popular tobacco company Weibo accounts, each of these had at least 5000 fans as of 31 December 2012. In addition, for tobacco corporations with more than one Weibo account, only one account would be selected which had a more obvious intention of tobacco marketing.

Of these ten Weibo accounts, three were excluded from the final study sample. One was created by ‘Mountain Ali Culture Communication Corporation’ in Taiwan. Although its description includes ‘Mountain Ali cigarette’, it had made only 147 posts with most describing the scenery of Mountain Ali. We also found two Weibo accounts set up by the Guangdong Tobacco Corporation. After examining posts to both of these accounts, we only selected the one titled as ‘Happiness together’ which had an obvious emphasis on tobacco advertising and promotion, and included a tobacco logo and brand name on its front page for inclusion in our study. This account had also received formal certification from the Sina Corporation. Weibo users can apply for ‘official certification’, which confirms that an account is truly operated by the organisation that is claiming to be behind it. This certification is helpful in winning trust from fans and increasing the number of fans. The third account excluded functioned solely as a company enterprise bulletin board for employees. After these three exclusions, seven Weibos related to Chinese tobacco company promotional activity remained in our study.

### Code book development

The full text of all posts made by these seven Weibos were downloaded by Rweibo which is a program that provides an interface to the Weibo open platform and allows textual analysis [26]. In total, there were 12,073 posts made by the seven tobacco company Weibos at the data collection date of 31 December 2012. In order to develop a code book for the content analysis of all the posts, a random sample of 5% of all posts (n = 600) was selected. First, two researchers (FW and PPZ) read through each post independently and used one word to describe the main meaning of the post. This produced a list of 70 words. Next, four researchers [FW, PPZ, DYY and HF] held a discussion to group the 70 words by combining the synonyms that were assigned to the 600 posts in to subthemes. A number of the words assigned were significantly overlapping in meaning and intent and a total of 14 subthemes resulted.

Third, the same four researchers combined the 14 subthemes into six main themes and clear definitions were developed and assigned to minimize overlap.

### Coding

Coding of all 12,073 downloaded posts was then performed by two trained staff who both independently reviewed all posts. Each post was classified into only one theme. In the case of disagreement between the two coders, a third coder was used to determine the final coding. To assess agreement between the two coders, a Kappa score of 0.89 was calculated which demonstrates that inter-coder reliability was excellent [27]. If the content of the post did not fit any one of the themes, it was coded as ‘other’ and was not included in the final analysis of this study. To identify what were the most frequently used words in the posts to gain insights into the marketing strategy of the tobacco companies, a word frequency ranking was conducted by Rwordseg. Rwordseg is an R environment open-source Java tool and one of the most accurate Chinese word segmentation tools [28].

## Results

Table 1 shows the detailed information of six main themes including traditional culture, popular culture, social and business affairs, advertisement, public relations and tobacco culture. The six themes were then classified into two categories: overt marketing and covert marketing. Overt marketing meant that a tobacco brand or product was being openly promoted, and covert marketing meant that the promotional message was linked to tobacco use, rather than directly mentioning a brand or product. Table 1 also illuminates how the original 70 words assigned to the 600 posts were collapsed into the two final categories of six main themes.

**Table 1:** Code book for this study.

Categories	Themes	Definition	Subthemes	Focus of Posts
Covert marketing	Traditional culture	A group ofphilosophies, art and history perspectives which are retrospected from ancestors.	Philosophy	Life philosophy, insights on life, famous quotes
			Literature & art	Serious literature, serious art
			History & geography	Chinese cultural history, cultural geography
	Popular culture	A kind of culture that originates from ‘the people’ which is also widely favoured or well liked by many people. [32]	Fashion	Clothes, ornaments, pop stars, cosmetics, decoration, entertainment events
			Leisure	Food, drinking, shopping, tourism, sports, healthy life, festivals custom, jokes
			Popular arts	Pop music, movie, TV, book and photography
	Social and business affairs	Including reports of social events and advice for business people.	Current event news	Environment protection, political affairs, economic events, social events
			Business advice	Time management, interpersonal skills, business etiquette
Overt Marketing	Advertisement	The introduction of tobacco brands and products through texts and images.	Tobacco Brand Introduction	Brand history, brand connotation, brand culture, multi-media brand dissemination
			Cigarette advertisement	Tobacco cultivation, leaves, production process, product additives, technique, taste, packaging.
	Public relations	The practice of managing the spread of tobacco information between tobacco industries and customers.	Online marketing	Guessing contests, Repost, lucky draws, favorite product recommendations, crosswords.
			Offline marketing	New products tasting, calls for slogans, club activities (tourism, sports, party, etc.), charity activities, culture contest (poems, photos, singing, etc.)
	Tobacco culture	The ways that tobacco industries associate tobacco products with history, literature and art to normalize smoking behavior in the daily life.	Cigarette use in daily life	Smoking manners, anecdotes related to smoking, celebrity smoking, smoking history and customs, tips for healthy smoking, smoking in classic literature, insights and experiences of smoking.
			Tobacco accessories	Holder, ashtray, lighters, snuff bottles, cigar knives.

Table 2 provides the basic information, such as the number fans, account name and date the account was opened, for the seven tobacco company Weibos. All seven Weibo account names included a tobacco brand. All of the accounts were established after January 2011 when Sina Weibo became the most popular microblog in China. Three of the Weibo accounts were set up by the tobacco companies directly, with the other four being established by marketing agencies. Detailed information about products were published in the posts through text, image and video files: from the growing environment of tobacco plants, the selection method of tobacco leaves, the production process, to the taste, the design of the package, as well as the brands' connotations.

**Table 2:** Basic information from seven most followed Chinese tobacco Weibo accounts, as at 31 December 2012.

Weibo Account Name	Brand Name	Number of Fans	Number of Posts	Account Registrants	Date Launched	Logo
Double Happiness Fashion	Double Happiness	252205	1612	*	2012/5/15	Trademark
Mountain Tai Club#	Mountain Tai	96510	3966	Shandong Mountain Tai Brand Culture Communication Co., Ltd.	2011/5/2	Trademark Commercial
Tongxian Manor#	Tongxian	33020	3050	Xiamen Tongxian Industrial Co., Ltd.	2011/9/15	Trademark Commercial
Zhongnanhai club	Zhongnanhai	25433	1531	Beijing Cigarette Factory	2011/1/11	Trademark Commercial
Happiness together#	Shuangxi	15826	429	Guangdong Shuangxi Culture Communication Co., Ltd.	2012/9/19	Commercial
Real Dragon Club#	Real dragon	11223	787	Guangxi Real Dragon Club Ltd.	2012/3/19	Trademark Commercial
Yuxi 1913	Yuxi	6577	698	**	2012/11/09	Trademark

# Tagged as ‘Official Weibo.’

*not shown in Weibo. However, the registered address was the same as the Shanghai Tobacco Corporation.

** not shown on Weibo. The content strongly suggests it may have been set up by the advertising agency employed by the Hongta Tobacco Corporation.

The ‘Double happiness fashion’ Weibo had the largest number of fans with 252,205, while ‘Mountain Tai Club’ published the most posts, 3966 as at 31 December 2012. Four Weibos were tagged as being ‘Official Weibo’, meaning that they had received the formal certification from the Sina Corporation, despite these accounts being used primarily for tobacco product promotion.

Among the 12,073 total posts made by the seven tobacco brands, 11143 posts (92.3%) could be classified into the six main themes shown in Table 3. Word frequency was calculated to provide possible insights into the communication objectives of the companies. Pleasant, attractive words were used to make the content of Weibos more friendly and appealing. For example, ‘wonderful’, ‘life’ and ‘beautiful’ were the most frequently used words in the posts under the theme of ‘traditional culture’ while among the posts under the ‘popular culture’ theme, the top three most frequently used words were ‘shopping’, ‘eating’ and ‘design’. In addition, content of different themes was presented in different ways. Posts under the category of ‘overt marketing’ included text, images and videos to present information about tobacco products and disseminate news about online and offline activities. Posts under the category of ‘covert marketing’ included content such as statements about philosophies of life, fashionable lifestyles, leisure and entertainment and social hot topics unrelated to cigarettes. These interesting and fashionable posts included a tobacco brand name as a Weibo topic tag. In all, ‘popular culture’ was the most frequently used theme, accounting for about half of all posts (49%), followed by ‘advertisement’ and ‘tobacco culture’ (both at 12%), ‘traditional culture’ and public relations' (both at 11%), and ‘social and business affairs (5%). Posts under category of ‘covert marketing’ accounted for 66% of all posts.

**Table 3:** Content analysis of the 11143 Weibo posts which were coded for the six main themes.

Marketing Strategy	Themes Percent Frequency (n)	Word Frequency Percent Ranking (n)	Illustrative Examples
Covert marketing	Traditional culture: 11% (1226)	Wonderful: 1.04% (n = 116); Life: 0.73% (n = 81); Beautiful: 0.38% (n = 42)	# Double Happiness Morning Post# While there is a will, there is a way. (a Chinese traditional proverb traced back to Han Dynasty)
	Popular culture: 49% (5460)	Shopping: 2.25% (n = 251); Eating: 1.80% (n = 201); Design: 1.04% (n = 116)	# Double Happiness creation # Aurora, a new glowing cocktail, can emit light in the dark, and looks like the aurora borealis.
	Social and Business Affairs: 5% (557)	China: 0.56% (n = 62); Job: 0.37% (n = 41); Communicate: 0.31% (n = 35)	# Mountain Tai · Current affairs reviews # To improve the environment of attractions in China, we should improve awareness of civilization.
Overt marketing	Advertisement: 12% (1337)	Cigarette: 7.88% (n = 878); Mountain Tai: 7.23% (n = 806); Tobacco Leaf: 1.15% (n = 128)	Smoke Mountain Tai cigarettes and appreciate the Confucian culture, hospitality and humility.
	Public relations: 11% (1226)	Activities: 4.92% (n = 548); Social gathering: 3.42% (n = 381); Gifts: 1.55% (n = 173)	#Real Dragon: What will you bring home in the Chinese New Year? Repost this and you will have opportunity to get real dragon New Year prizes!
	Tobacco culture: 12% (1337)	Cigarette: 16.0% (n = 1783); Smoking: 8.61% (n = 959); Pipe: 1.12% (n = 125)	# Mountain Tai • Feeling of smoking # Thinking about the early years when cigarettes are rarities. Cigarettes were symbols of the people's identity.

Among the seven Weibos selected for this study, the different tobacco companies all linked tobacco use to content that was appealing to youth and young adults (see Table 3). As illustrative examples show in Table 3, Weibo fans were encouraged to strive towards better life under the theme of traditional culture. Tobacco use was connected to living a fashionable, trendy life style via posts containing links to fashion news and content.

Table 4 shows that different tobacco brand Weibos adopted different marketing strategies. For example, the proportion of posts under popular culture accounted for 67% for ‘Double Happiness’ but only accounted for 10% in ‘Real Dragon Club’, where public relations accounted for 32% of the total posts.

**Table 4:** Comparison of proportions of the themes in different Weibo (%).

Weibo Name	Traditional culture	Popular Culture	Social and Business Affairs	Advertisement	Public Relations	Tobacco Culture
Double Happiness Fashion	10	67	4	11	7	2
Mountain Tai Club	11	23	6	16	3	41
Tongxian Manor	35	29	6	12	13	5
Zhongnanhai club	0	32	1	8	46	12
Happiness together	5	25	1	8	60	1
Real Dragon Club	0	10	0	26	32	31
Yuxi1913	18	43	12	10	9	8

The results also show that 33% of posts included the word ‘cigarette’ or ‘smoking’ and 53% posts included a tobacco brand name either as a topic tag or in the text of the posts. In addition, over 68% of posts had topic tags and 79% were original posts as opposed to re-shared posts.

We also found some posts focused on emotional arousal to encourage smoking such as ‘When you are alone in the dark night with no lights, you need a cigarette to light your life’ (from Tongxian Manor) and ‘Cigarettes mean sincerity, warmth and sharing’ (from Mountain Tai Club). Other posts included statements to try to minimize the harms of smoking. For example, advice under the topic tag of ‘smoking and health’ encouraged tobacco users to do more exercise and use traditional Chinese medicines to reduce the harms of smoking. Also, some posts tried to divert audience attention away from smoking harms to other health risks. The ‘Real Dragon Club’ shared content under the topic ‘environment and health’ that included claims that ‘air pollution is the most important factor leading to lung cancer, not smoking’.

The strategies adopted by the tobacco companies on Weibo were successful in generating interaction with fans. For example, the ‘Zhongnanhai’ brand published a post urging the public to participate in the ‘Capital Charity Program’ which helps teenagers to realize their dreams. This post was re-posted 330 times and commented on by 167 Weibo users.

## Discussion

### Main findings

This study demonstrates that several Chinese tobacco companies have embraced Weibo to open dialogue and build relations with target customers by exploiting a major loophole in the current tobacco advertising laws. Given that new media are quickly superseding traditional media as an efficient tool for the dissemination of information and persuasive marketing promotions, this study provides sound evidence that regulation to ban Internet advertising of tobacco in China is urgently needed.

Weibo users are primarily youth and young adults, with 54% of users being under 30 years of age [14].

While our study found that all the tobacco companies linked smoking to youth- friendly content, there were also different marketing strategies adopted according to the particular brand culture and target consumer. For example, Double Happiness, a brand favoured by young people, focused its posts on popular culture. For the ‘Mountain Tai Club’ Weibo, a brand named after an actual mountain in Shandong Province, homeland of the Chinese philosopher Confucius, nearly half of all posts focused on tobacco culture.

The Weibo posts not only helped to establish a positive image of the tobacco industry, but were also constructed to help normalize smoking behaviors. Tobacco advertisements and promotional content were mixed in with information about food, sports and social events. Positioning tobacco use within these commonplace topics conveys that smoking should be considered a normal part of everyday life. Such tactics could significantly weaken the positive impact of tobacco control policies. In general, the tobacco industry made efforts to normalize tobacco use through both perceptual and rational ways. As shown in this study, some tobacco industry accounts utilized emotional arousal to normalize tobacco use, while other accounts sought to minimize the health effects of smoking. Such descriptions not only potentially weaken knowledge of the harms of tobacco use, but also positively reinforce smoking behavior. Our results confirm that Weibo offers the Chinese tobacco industry an efficient channel to rapidly spread messages about their products and normalize smoking behavior.

### Implications for policymakers

With over 300 million users, Weibo is now a major site for discussion of all aspects of contemporary Chinese life [29]. Weibo presents an unparalleled opportunity for the tobacco industry to keep tobacco products in front of current and potential customers without any of the constraints of tobacco advertising legislation. Because new media reflects current social issues and helps establish social norms, monitoring tobacco marketing on new media should be standard practice in tobacco control.

The WHO FCTC also provides impetus for policy makers to take action to end tobacco industry promotions on Weibo. According to the WHO FCTC Article 5.3 implementation guidelines, which focus on the protection of public health policies from tobacco industry interests, signatory countries should require that any product information provided by the tobacco industry be transparent and accurate [30]. Additionally, as per WHO FCTC Article 13, a comprehensive ban on tobacco advertising should include not only traditional media, but also new media platforms including social media.

Also, as internet communication can easily cross borders and influence people from other countries, monitoring will require international cooperation. Countering online tobacco industry marketing through effective social media campaigns is a promising area of tobacco control activity [31].

### Limitations

Several potential limitations of this study need to be addressed. First, this study only analyzed 92.3% of the total sample that could be classified into the six main themes. However, the 930 ‘other’ posts may contain material that contradicts or is different to the main results. In addition, though we analyzed both original posts and re-shared posts from the tobacco industry, we did not examine why 21% of posts were re-shared by followers and other users.

### Future study

This is a descriptive study which provides information on the Weibo accounts established by the tobacco industry. Further studies could be conducted to assess the possible impact of the posts on both smoking and non-smoking Weibo users. Social media platforms continue to change, evolve and innovate and tobacco control researchers must keep up with emerging technology and communication vehicles. For example, since completing this study, Wechat, a mobile text and voice messaging communication service in China, has gained a huge audience market share and could be another potential source of tobacco marketing exposure.

## Conclusion

The Chinese tobacco industry has skillfully employed youth-oriented themes to promote their brands and products and normalize smoking. These promotions expose serious gaps in existing tobacco advertising laws that do not include online media. Given the rapid increase in internet users, more effective measures in accordance with WHO FCTC Article 13 should be adopted to monitor and regulate the online marketing efforts of the tobacco industry.

**Provenance and peer review:** Not commissioned; externally peer reviewed.

**Data sharing statement:** The unpublished data from this study, including the records and pictures, are available by contacting PPZ (email: zpinpin@shmu.edu.cn; telephone: 86-21-54237202). Data can be sent by email.
